# The Apple Bites Back: Claiming Old Orchards for Residential Development

**DOI:** 10.1289/ehp.114-a470

**Published:** 2006-08

**Authors:** Ernie Hood

As the U.S. population continues to grow, increasing demand for housing and related community resources means more land is being converted from agricultural uses to residential applications. According to the revised 1997 National Resources Inventory conducted by the USDA Natural Resources Conservation Service, more than 6 million acres of American farmland were converted to developed uses between 1992 and 1997. That is an annual conversion rate of roughly 1.2 million acres per year—a 51% increase over the average annual rate reported for the preceding decade.

Naturally, many of these areas were routinely treated with pesticides and other chemicals during their agricultural lifetimes. Although this legacy has been problematic in a wide variety of land conversion scenarios, one in particular seems to have attracted the attention and concern of environmental officials and property buyers in several states across the country: the residential development of historic orchard properties. In state after state, these old orchards (which most often produced apples, but also peaches, cherries, pears, and other tree crops) are metamorphosing into highly desirable subdivisions—desirable, that is, until it emerges that the soil beneath the feet of the proud new residents may be contaminated with lead and arsenic. These toxic by-products are left from the days before DDT and before organophosphates, when arsenical pesticides, particularly lead arsenate (LA), were the treatment of choice to prevent the ravages of insect damage.

## They Loved LA

LA was introduced in 1892 in Massachusetts for use against the gypsy moth. Two other arsenical pesticides (copper acetoarsenite, known as “Paris green,” and calcium arsenate) also were in use, although LA largely replaced them in the 1930s due to lower cost, greater efficacy, and lower phytotoxicity. Even though arsenic residue was recognized as a problem as early as 1919, LA was the most widely used pesticide in the nation—recommended by the USDA and applied to millions of acres of crops—until the late 1940s, when DDT (considered at the time to be safer and more effective) became available. LA continued to be used in some locations into the 1970s, and was ultimately banned in 1988.

LA was perhaps most commonly applied in apple orchards, due to its excellent control of the codling moth, a major apple pest. Today, apple orchard properties that were in production during the heyday of LA use are the focal point of environmental concerns; given the nature of the pests peculiar to orchard crops, growers tended to apply the chemicals frequently and in high concentrations, often over many years. “In some cases, they dusted the apple trees or peach trees every week, whereas most field crops may have had one or two applications during the growing season,” says Kevin Schick, a bureau chief with the Site Remediation and Waste Management Program in the New Jersey Department of Environmental Protection.

LA and the other arsenical pesticides were designed to be persistent, and it is that persistence that is causing environmental contamination problems decades after their use ended. “These chemicals have just tremendously long half-lives in the ground,” says North Carolina state toxicologist Ken Rudo. “They bind very tightly to the soil.”

Once LA reached the soil through over-spray, spillage, rainfall wash-off, or simply fallen fruit and leaves, the lead arsenate underwent hydrolysis, separating into lead and arsenic bound to organic particles in the soil. The lead, being poorly soluble, was immobilized, typically within the top 12 to 18 inches of topsoil. The fate of the arsenic was similar, but a bit more complicated. “Arsenic, as arsenate, even though somewhat sparingly soluble, *is* soluble, and it will move in water,” says Washington State University soil scientist Frank Peryea. “I’ve seen some sites where almost all of the arsenic is still in the topsoil, in the tillage zone, and I’ve seen sites where I’ve measured arsenic movement as deep as a meter or so.”

Carl Renshaw, a hydrogeologist at Dartmouth College, published a study in the January/February 2006 issue of the *Journal of Environmental Quality* showing that arsenate in the soil can be remobilized by being disturbed. He compared two fields in the same historic New Hampshire orchard. One field had never been disturbed, whereas the other had been tilled and replanted in the early 1990s. “What we found was that in the field that had been replanted, there was somewhat less arsenic on it than in the undisturbed field,” he says.

Given the assumption of virtually identical application rates over the years, the discrepancy apparently arose from a portion of the arsenic in the disturbed field having been mobilized and removed by surface water. Renshaw found arsenic in the sediment of a nearby stream in amounts that very closely matched the arsenic missing from the tilled field.

“The implication from our study,” says Renshaw, “is that if you’re not really careful about erosion, you’re going to end up sending a lot of arsenic down into the stream channel.” To date, researchers have seen no evidence of direct health effects in humans, animals, or plants exposed to this stream-bound arsenic. However, more study is needed to fully understand the ramifications—if any—of the mobilization.

## How Dangerous?

The potential danger posed to human health by lead and arsenic contamination in historic orchards is a complex issue, fraught with scientific uncertainties and competing interests. Arsenic is a known human carcinogen. Exposure to lead, especially prenatally and in childhood, can lead to neurological damage. There is no doubt that excessive exposure to either substance can adversely impact health, but in this case any risks are almost exclusively long-term—virtually no instances of acute adverse health effects have been documented in people living on historic orchard properties.

Regulatory agencies such as the EPA and state health and environmental departments determine allowable levels of chemicals in soils and water based upon formulas that take into account criteria such as toxicity, exposure, and naturally occurring background concentrations of the chemicals. For carcinogens such as arsenic, the calculations are based upon the amount of a chemical that is predicted to result in 1 additional cancer case occurring in 1 million people exposed over their lifetimes. But there is some flexibility in the standards based on local conditions and practical considerations. In New Jersey, for example, where background arsenic concentrations are often high, the criterion for residential soil cleanup is set at 20 ppm—50 times the EPA’s level of 0.4 ppm.

In historic orchard properties, cleanup action is often triggered when a so-called “hot spot” is discovered—typically an area where the pesticides had been mixed and loaded or stored, and where repeated spills or disposal of excess materials may have occurred. The contaminant concentrations in those hot spots can be significantly higher than in the tree crop areas. But locating hot spots after many decades can be very difficult.

The ATSDR is often called in to analyze the health risks at contaminated historic orchard properties. “We look at the contaminants, the concentrations, the pathway, how long [residents] are exposed to it—all of the different aspects of an exposure,” says Robert Safay, an environmental health scientist with the agency. “For example, when you’re looking at lead contamination in the soil, you’re primarily concerned about young children playing out in the soil.”

In all but the most extreme cases, the health risks of living atop contaminated historic orchard soil are ultimately characterized as very low and manageable. Exposure is the critical element. “The real issue here is direct contact—you want to limit the direct contact,” says Lori Bowman, director of the Agrichemical Management Bureau in the Wisconsin Department of Agriculture, Trade, and Consumer Protection. As Safay explains, there must be a completed exposure pathway for there to be even the potential for health effects. Ultimately, the amount of risk depends on the level of contamination and the use of the land.

For the most part, residents are advised to limit their direct exposure to the soil if it’s unremediated and to take simple measures such as wearing gardening gloves and wiping their feet before entering the house. Peryea says there is little risk from eating plants grown in this type of soil, but advises that home gardeners rinse off produce before bringing it into the home, then wash it again with a detergent and scrub brush to remove any remaining soil particles, paying particular attention to rough vegetables like broccoli and leafy vegetables like lettuce, which can trap and retain dust. He also advises paring root and tuber crops such as potatoes, carrots, and radishes, and not composting the peelings or other unused plant parts.

The risks involved may be modest and long-term in most cases, but low risk is not the same as no risk, and regulatory agencies across the country are finding themselves in a thorny situation as more and more contaminated historic orchard properties are developed. They are caught between their duty to protect public health and the environment, and the fact that the risks presented by most of these properties pale in comparison to those associated with other, more acute contamination sites, such as lands near smelters or toxic waste dumps. Naturally, budgets are limited, and priorities must be set. Yet the orchard situation cannot be ignored, and several states have been wrestling with how to deal with this issue for several years.

The sheer scope of the phenomenon adds another layer to the challenge of how to most effectively deal with it. “The magnitude of the problem is just staggering,” says Peryea. Millions of acres across the nation are involved. In the state of Washington alone, Peryea says, some 188,000 acres are affected. In Wisconsin, 50,000 acres may be affected, and in New Jersey, up to 5% of the state’s acreage is estimated to be impacted by the historical use of arsenical pesticides. Both New Jersey and Washington have had multistakeholder task forces examine the problem and issue recommendations and guidelines.

Wisconsin is likely to convene a similar task force later in 2006, according to Bowman. “We want to develop a protective, economical, and practical strategy to address potential residues of lead and arsenic in soils related to historic orchard use,” she says. “The charge of the task force would be to evaluate the health and environmental impacts, and [also evaluate] what kind of alternatives and strategies we could put into place to limit exposure and to educate and provide outreach to homeowners and developers as to what types of precautions can be taken at these orchard sites to mitigate any risk.”

## What Can, Should, or Must Be Done

Because contamination can be spread over large areas, remediation measures vary widely, depending upon the level of contamination, the current or intended use of the property, and state or local regulations. Each method has its advantages and its drawbacks, and each site has its own unique circumstances that will often dictate how, when, and even if the situation will be dealt with.

Excavation is the quickest and most thorough remediation method. This involves scraping up the contaminated topsoil, hauling it away to an approved landfill, and replacing it with clean dirt. Realistically, says Peryea, removal is the only way to eliminate risk, “but it’s very expensive.” Such total remediation can cost $1 million per acre or more. And it’s a huge undertaking. Peryea does the math for 1 acre: “If you have contamination down to three feet, you’re looking at getting rid of three acre-feet of soil—that’s twelve million pounds of soil.”

Capping, which involves simply putting a 12- to 18-inch layer of clean soil over the contaminated soil, has been used in some locations. However, this requires enormous amounts of clean dirt. Further, capping cannot be considered a permanent solution—plants will grow on the soil caps, their roots will penetrate the contaminated soil, and the vegetation will eventually redistribute the lead and arsenic to the clean soil. Also, it is common for the soil caps to be disturbed by construction activities.

Soil blending is another alternative, and one that is growing in popularity, particularly when contaminant concentrations are only minimally in excess of actionable levels. This involves bringing clean soil to a site and mixing it with the existing topsoil, with the intent of reducing concentrations below levels that require health-protective actions. Although relatively effective, blending can be a hit-or-miss operation. The main reason is that operators can’t always achieve 100% blending, and it very much matters where the subsequent samples are taken—even a few inches can make a difference. Sometimes it is necessary to repeat the procedure, which, of course, drives up costs. Also, disturbing the soil in this way could actually mobilize the arsenic, as Renshaw’s research showed. Regardless of its shortcomings, however, blending is an option many states have chosen in recent years.

In some instances, a simple solution can be adequate. “What seems to do a good job of reducing exposure in areas where people aren’t digging in the soil is just to keep turf on it, or keep it vegetated somehow,” says Peryea. At some sites, simply moving the contaminated soil to another location on the site and capping it—for example, by burying it under a roadway—has been acceptable, although this option requires that a deed notice be executed, so that all of the records of the sampling and disposal of the contamination become part of the property’s permanent title record.

Thus far, other remediation methods have proven to be ineffective, impractical, or counterproductive on these sites. Researchers such as David Butcher, a professor of analytical chemistry at Western Carolina University in Cullowhee, North Carolina, have explored the possibility of phytoremediation of these properties, in which plants are used to suck the contaminants out of the soil, after which the contaminated biomass is destroyed. But this method, though effective in certain remediation situations, doesn’t appear to hold much promise in lead- and arsenic-contaminated orchard soils. Phytoremediation is quite slow, potentially taking decades or longer to effectively remove contaminants. Butcher also was unable to discover a method of removing the lead from the soil without the addition of other chemicals (such as EDTA) to release the tightly bound element.

One way to release the lead is by adding phosphorus to the soil, but this also mobilizes the arsenic. “That creates an even bigger problem,” Peryea says. “If you get the arsenic moving, and it moves down into the ground-water, cleanup becomes much more difficult than trying to keep it in the topsoil.”

According to Peryea, you can scratch microbial volatization as well. In that method, native soil microorganisms are stimulated to volatilize arsenic. The gaseous arsenic can then be trapped. But for this method to be effective, soils must be kept quite wet. Many of the historic orchard properties are well-drained, sloping sites, where it would be difficult to keep the soil adequately flooded. Plus, of course, as Peryea points out, “if you are evolving arsenic off your soil, and it flows down and contaminates your neighbor’s property, that’s going to create some problems.”

Cleanup and real estate disclosure issues are usually handled at the state and local levels, where approaches vary considerably. As public awareness of the potential contamination of historic orchards increases in the affected areas, state agencies are fielding more and more calls from concerned property owners or prospective buyers. Chuck Warzecha, a risk assessor with the Wisconsin Department of Health and Family Services, fields 10 to 15 such calls a year. He tries to give concerned citizens a balanced message. “My first statement is that it’s not a real scary issue and doesn’t have to be a big problem on their property,” he says. “It’s something that now that they know about it, it’s worth doing something about, but they shouldn’t be concerned that past exposure is going to be a real serious issue for their families.”

If callers haven’t had their soil tested yet, Warzecha recommends that they do so. Then he advises them on how to manage the problem if there is one. If contamination hot spots are identified, cleanup may be required under Wisconsin’s Agricultural Chemical Cleanup Program. In such cases the property owner would pay a 25% deductible, with the rest of the costs covered by the state, according to Bowman.

In Washington, the Model Toxics Control Act requires the reporting, study, and cleanup of sites where hazardous substances are above state-set cleanup levels. In residential developments, the state is working to increase awareness of the potential for contamination on historic orchard lands, particularly among developers. The goal is to get developers to incorporate that consideration at the outset of projects, when there are opportunities to deal with problems more easily than could be done once housing is in place. As in other states, several departments are involved in providing consultation, health assessment, and technical assistance on a case-by-case basis.

Washington has also chosen to be proactive in its cleanup efforts at sites where children are especially likely to be affected. “We have elected to focus on schools, child care facilities, and parks where groups of young children might be present, trying to take steps to reduce exposures for kids,” says Dave Bradley, a toxicologist and risk assessor with the Toxics Cleanup Program in the Washington State Department of Ecology. “We’ve focused on a handful of counties, and have further focused on schools, trying to integrate with existing community processes such as school construction, and then trying to prioritize how we use either our authority or funds out of the state Superfund to actually perform some of the cleanup actions.”

In New Jersey, the recommendations and guidelines put forth in the 1999 report of the Historic Pesticide Contamination Task Force set the agenda. Schick, whose department handles historic orchard contamination cases, says there’s no excuse for ignorance on the part of New Jersey developers at this point, and it should be a standard element of their due diligence.

“It’s common knowledge, the guidance is out there, it already involved the real estate agents, the bankers, the insurers, the farm bureau,” Schick says. “It’s been out there long enough that anyone making any kind of investment in developing farmland should have known about it, and they will be held at fault for not coming to the department or cleaning prior to development.”

## Paradise Lost, Paradise Regained?

Today, Barber Orchard, a 500-acre subdivision located a few miles west of Waynesville, North Carolina, is “not a place where it looks like there are any problems,” says Butcher. “It’s not a place like where there’s been a lot of mining and it looks like a moonscape. It looks beautiful up there.” It may look beautiful, but that doesn’t change the fact that Barber Orchard has had a troubled history.

Barber Orchard was a commercial apple orchard from 1903 until the mid-1980s, when the operation went bankrupt and the land was parceled off for development. In 1999, a pregnant resident heard rumors of birth defects from neighbors and friends in the area. She contacted Rudo, who, with the county health department, initiated an extensive investigation that included soil and water sampling and a series of public meetings with residents. In late 1999 through mid-2000, the federal EPA conducted a $4 million emergency removal of a foot of topsoil from 28 residents’ yards.

Reflecting the tremendous variation in contamination typical of historic orchard sites, the EPA found only trace amounts of lead and arsenic in some sampling locations, but several others were well in excess of the agency’s cleanup goals of 40 ppm arsenic and 400 ppm lead. Samples came in as high as 400 ppm arsenic and 1,200 ppm lead. The highest levels were detected at spots where trees were still located, or had been cultivated in the past, reflecting the cumulative impact of long years of pesticide applications.

In 2001, the site was placed on the National Priorities List under the Comprehensive Environmental Response, Compensation and Liability Act (CERCLA), an unusual step for a historic orchard. “CERCLA authority is hobbled when it comes to normal use of pesticides,” says James Bateson, branch head of the Superfund Site Evaluation and Removal Branch of the North Carolina Department of Environment and Natural Resources. “In cases where [a pesticide has] been spilled or dumped in large quantities or misused, that’s when CERCLA can have some authority. At Barber Orchard, the case was made that there was enough spillage associated with the way they handled things up there that it wasn’t normal application of pesticide.”

“The way they handled things” was by distributing the pesticides through a unique underground high-pressure piping system, with aboveground nozzles at the tree sites where sprayers were hooked up. The system left pesticide hot spots at several locations throughout the orchard property. “If there was spillage at a particular location above-ground where that particular distribution pipe was located, or if there was a fracture in the pipe, or a joint in the pipe that got a crack or leak in it, then we may have contamination locally at that one particular site, or along the connections along the way,” explains Haywood County Health Department director Carmine Rocco. According to Bateson, the EPA has in fact found several places where pesticides had leaked into the soil because of poor maintenance of the piping system.

In 2004 the EPA issued a record of decision (a document specifying how the agency planned to clean up the site) for the orchard’s soil, calling for much more removal of contaminated dirt, mainly from vacant lots on the property. “What we’re doing right now is waiting for funding to implement the cleanup for soil,” says Jon Bornholm, the EPA’s project manager for the Barber Orchard site. That phase of the cleanup, which should take less than a year, is projected to cost $20 million, and there’s no telling when the funds will be released by the EPA for it to take place.

The EPA is expected to render a record of decision for dealing with groundwater contamination on the site before the end of 2006. Bornholm expects that the agency will opt for “monitored natural attenuation”—in other words, let Mother Nature take care of the problem, and hope that contaminant concentrations will decrease over time through natural processes such as biodegradation and dispersion. He guesses that could take 30 to 50 years, with the EPA monitoring the situation continually. Residents have been advised to filter their well water since the problem was uncovered, and city water is now available to the site, although not all of the current homeowners have elected to hook up to the service.

Since the problem arose, the ATSDR has also been involved at Barber Orchard, evaluating the health situation. In April 2002, the agency released its official public health assessment for the site, which concluded that “current exposures to site contaminants are not likely to result in adverse health effects. . . . The exposure pathways for lead and arsenic were disrupted within a relatively short time frame, so past exposures are not likely to lead to health effects at this time.”

Meanwhile, Barber Orchard’s tax values have increased, and buying and selling of homes in the subdivision has not been hurt by the site’s Superfund status. “The heat of the moment has passed, and I think we’ve gotten over the panic mode,” says Ellis Morris, president of the Haywood County Board of Realtors. “Initially, people were tentative about buying in to that particular neighborhood, but that’s been resolved, there’s a comfort level now, and the real estate there is keeping pace with all of the other areas of Haywood County in terms of days on the market and selling price.”

David Miller would agree with that assessment. He and his wife retired to Barber Orchard from Florida in 1997, and his 1.4-acre lot was one of the properties cleaned up by the EPA. He is unconcerned about the contamination at the site and thinks the whole situation has been overblown. “I haven’t changed the way I live,” he says. “I work in the garden just about every day, I’ve planted a vegetable garden and eaten the vegetables, I’ve planted some fruit and eaten the fruit. So it has not affected me or my wife in any way.”

So it appears that Barber Orchard was paradise lost for a time, but is now paradise regained. Now, however, some neighbors just down the road may be facing a similar situation. In May 2006 residents of the Tan Woods and Orchard Estates subdivisions, built on what was once Francis Orchard, were notified that soil samples from a vacant lot at the site had tested positive for lead, arsenic, and other pesticides—a mix similar to that found at Barber Orchard. And like Barber Orchard, Francis Orchard was equipped with an underground pesticide piping system.

It’s still early in the process, and the results of more thorough sampling and testing are not yet available, so it’s too soon to predict whether Francis Orchard may eventually become a Superfund site. But this time around, according to Bateson, both residents and involved officials can benefit from the Barber Orchard experience. At Francis Orchard, he says, “the residents are well schooled after seeing what’s gone on at Barber Orchard, and of course the county and state people have been around the block now too.”

## Questions Remain

Despite the large scale scope of the problem, it appears that living on a historic orchard property contaminated by lead and arsenic does not constitute an immediate threat to human health. So it is still an open question whether it’s really necessary to spend huge amounts of money, often from tax dollars, to ameliorate these sites.

Peryea thinks that what is needed is a solid epidemiologic study to document whether there really is a problem with people living on these arsenical pesticide–contaminated soils. “If that sort of study was done,” he says, “and it was to show that there’s no problem, or that the problem is controllable by setting up some sort of engineering controls or behavioral controls, like they do with urban lead nowadays, that would probably take care of a lot of the problem. The response—rather than trying to force a cleanup that would probably be wildly impractical, very expensive, and potentially ruin property values—would be that people would change their behavior a bit and end up minimizing the risk.”

## Online Resources

New Jersey, Washington, and Wisconsin offer detailed advice to residents, developers, and other interested parties about what to do if they suspect or know their land is contaminated. Wisconsin has posted a variety of publications (**http://www.datcp.state.wi.us/arm/agriculture/pestfert/pesticides/accp/lead_arsen_resources.jsp**), including tips for safe gardening in lead- and arsenic-contaminated soil. Washington provides a comprehensive toolbox of resources stemming from its Area-Wide Soil Contamination Project, a task force that addressed not only historical orchard contamination, but also lead and arsenic contamination over widespread areas of the state from smelters and leaded gasoline combustion; see **http://www.ecy.wa.gov/programs/tcp/area_wide/area_wide_hp.html**. New Jersey offers the report of the Historic Pesticide Contamination Task Force (**http://www.state.nj.us/dep/special/hpctf/index.html**) and i-MapNJ, an environmental mapping tool that lets residents obtain detailed contamination information for specific locations (**http://www.state.nj.us/dep/gis/depsplash.htm**).

## Figures and Tables

**Figure:**
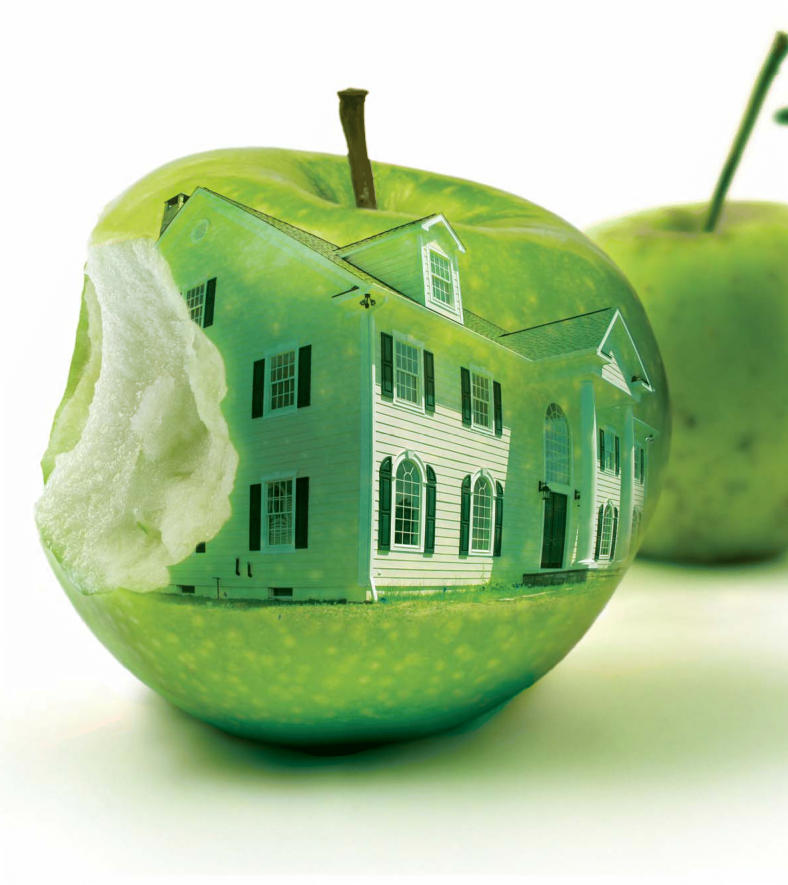


**Figure:**
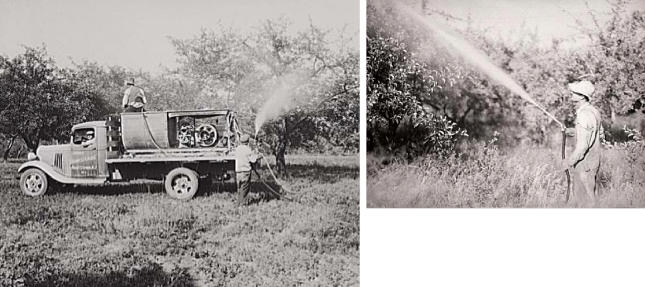
You spray, you pay? Spraying of arsenical pesticides on apple orchards was routine from the late 1800s through the 1940s. Lead arsenate was not banned, however, until 1988.

**Figure:**
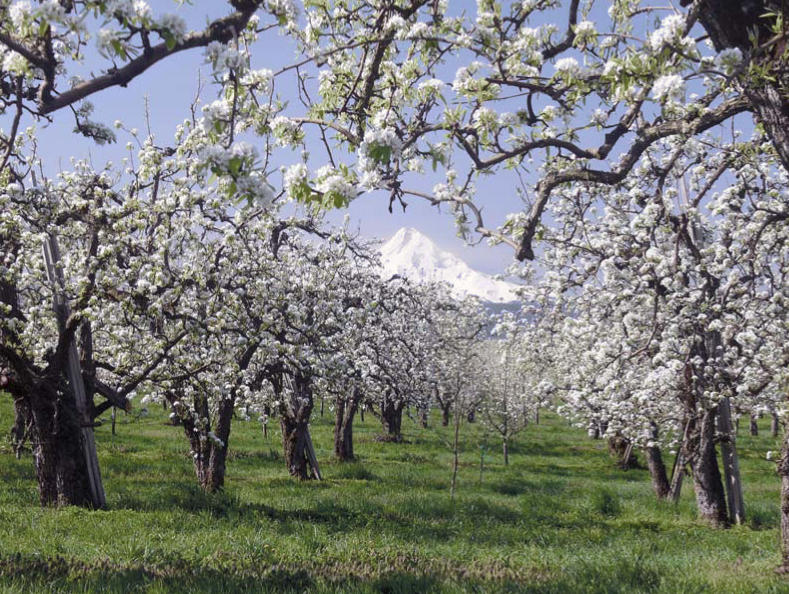
A blooming problem? Land that was once home to fruit orchards is now being turned into subdivisions, raising questions about pesticides that may still be present in the soil and the potential risks they pose to residents.

**Figure:**
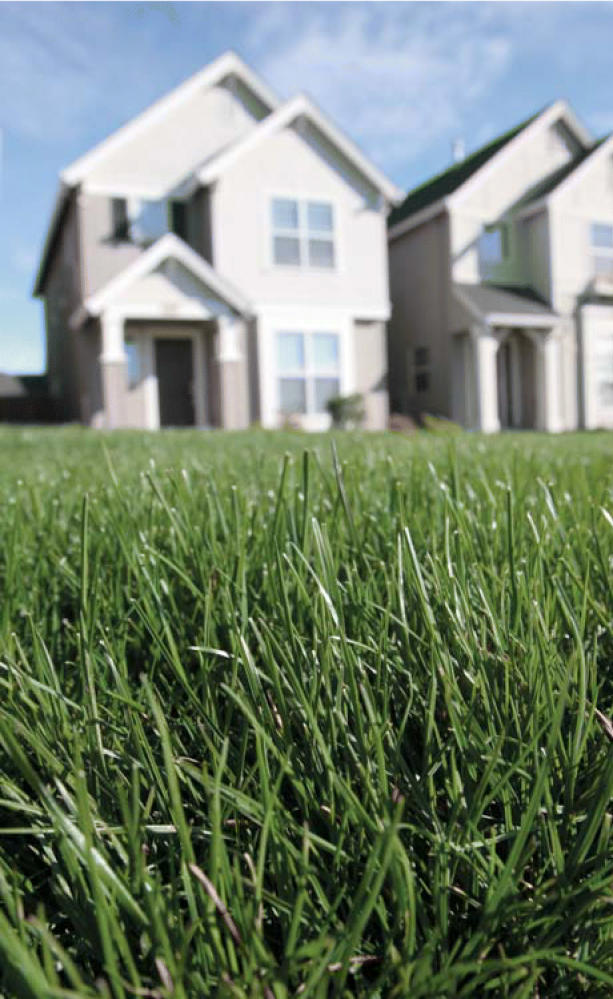
Soil survivors Some experts recommend that homeowners have their soil tested for arsenic and lead, although no perfect method exists for remediating soil that is found to still be contaminated.

**Figure:**
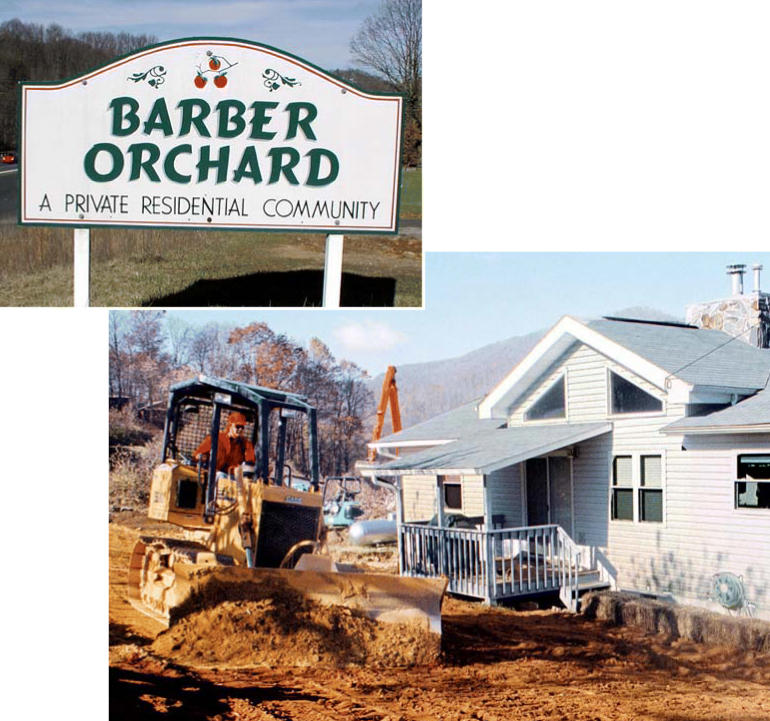
Landscraping A bulldozer scrapes a layer of contaminated soil from a yard in the Barber Orchard subdivision in North Carolina, once the site of a large apple orchard. Due to contamination with former agricultural chemicals, the subdivision was designated a Superfund site in 2001. EPA-supervised cleanup, mainly by removing soil, is on hold pending further funding.

